# Special Issue “State-of-the-Art Molecular Plant Sciences in Japan”

**DOI:** 10.3390/ijms25042365

**Published:** 2024-02-17

**Authors:** Setsuko Komatsu, Matsuo Uemura

**Affiliations:** 1 Faculty of Environmental and Information Sciences, Fukui University of Technology, Fukui 910-0028, Japan; 2 Faculty of Agriculture, Iwate University, Morioka 020-8550, Japan

Food shortages are one of the most serious problems caused by global warming and population growth in this century [1]; consequently, it is important to increase food production. Climate change influences the magnitude/frequency of hydrological fluctuations, high/low temperature, light quality, and so on, creating an unfavorable environment for the growth and development of crops [2,3,4]. The food self-sufficiency rate is 37% on a calorie basis; conversely, 63% of food of Japan is dependent on imports, although the value of agricultural imports increased by 31.2% in 2022 from the previous year [5]. To achieve a stable supply of food, it is necessary to efficiently promote the expansion of production in Japan of highly overseas-dependent items such as wheat, soybeans, feed crops, vegetables, and so on.

Plant scientists in Japan have been working to understand plant performance under fragile, unexpectedly but seemingly consistently changing environments, which will contribute to developing plants that are more resilient to the changing climate. This includes understanding the genetic basis of stress tolerance and employing advanced breeding techniques to create plants beneficial to humans that can thrive under altered environmental conditions [6,7,8,9,10]. Researchers are investigating how climate change affects plant metabolism, including processes such as photosynthesis, respiration, development, and nutrient uptake, as well as communications of plants with surrounding biological communities. Understanding these mechanisms is crucial for predicting how different plants will respond to a shifting climate.

This Special Issue aims to provide a comprehensive overview of recent advances in plant molecular science in Japan by inviting contributions from Japanese research institutes/laboratories that consolidate our understanding of this area. Potential topics regarding molecular studies in plants include biophysics, biochemistry, molecular biology, cell biology, developmental biology, synthetic biology, computational biology, omics, bioactive phytochemicals, plant–microbe interactions, pests/diseases, and the development of new technologies in plant sciences. This Special Issue published in the International Journal of Molecular Sciences consists of six contributions, including five original research articles [11,12,13,14,15] and one review [16].

Because the accurate analysis of a large number of transcripts, proteins, and metabolites promotes the knowledge of biological systems, omics such as transcriptomics, proteomics, and metabolomics has been performed on agricultural materials [17]. Based on the results from omics analyses, the roles of key factors in plants related to stress tolerance were carefully confirmed with molecular biological techniques [18]. “Multi-omics” application supported by genomics, transcriptomics, proteomics, and metabolomics has been quite useful to investigate and understand the biochemical, physiological, and molecular aspects of plants under environmental stress conditions [19]. In this Special Issue, four articles among five original articles are using omics techniques, such as proteomics [11,12] and transcriptomics [13,14]. These approaches contribute to not only understanding biological mechanisms in crops, but also producing crops.

Watanabe et al. [11] studied the precise effects of feeder cells and auxin on the growth and development of rice using an in vitro fertilization system. This study indicated that hydrolytic enzymes released from feeder cells are involved in the progression of rice zygotic development. Komatsu et al. [12] reported the usefulness of fiber crosslinked with zinc-oxide nanoparticles to develop a more effective method using nanoparticles for the enhancement of soybean growth. They concluded that fiber crosslinked with zinc-oxide nanoparticles enhanced soybean growth through the increase in photosynthesis/secondary metabolism and the accumulation of NADPH oxidoreductase, which is related to the effect of auxin. Xiao et al. [13] elucidated the effects of light quality on metabolism and gene expression in tomato fruit. They indicated that the altered gene-expression level encoding metal ion-binding proteins, metal-tolerance proteins, and metal transporters in response to blue and red light changed in the ionomic profiles of tomato.

Takase et al. [14] analyzed the expression profiles of several flowering-related genes in gentian plants. Particularly, they focused on the expression of transcription factors at different timepoints of the day. They found that the expression profiles of flowering-related transcription factors such as *BBX* and *MADS-box* families were different, but clustering analysis revealed that the expression of transcription factor genes were overlapped with that of *GtFT1*. Salam et al. [15] investigated the effect of insecticides on non-target communities, especially on endophytic bacterial communities, to understand how plant–endophytic bacteria interactions, which are beneficial to plant life under both normal and challenging conditions, are influenced. They found that insecticide use negatively affected non-target endophytic bacterial communities and, interestingly, plants can regulate and moderate their microbiome during their lifecycle depending on surrounding environmental conditions.

Heat stress negatively affects growth and development by inhibiting various physiological traits of crops [20]. It is known that mitochondria, chloroplasts, and cell membranes are particularly sensitive to heat stress [21,22]. On the other hand, targets of rapamycin (TOR) and SNF-related protein kinase 1 (SnRK1) are known to play important roles in switching signals underlying growth, development, and stress responses depending on energy status [23,24,25,26]. Suzuki et al. [16] reviewed that the possible contributions of TOR and SnRK1 to the heat responses of plants are supported by the integration of TOR and SnRK1 with mechanisms involving heat-shock transcription factors, alternative oxidase, and reactive-oxygen species production as well as functions of chloroplasts and mitochondria.

Considering the plant physiology research conducted globally, the research fields covered in the original papers included in this Special Issue overlap with those that many researchers focus on to understand fundamental mechanisms and explore applying findings to applications in agriculture to improve the quality of life of human beings. For example, the development–phytohormone relationship in embryogenesis (covered by Watanabe et al.) has been extensively studied [27,28,29]. This is also the same in the research areas covered by Komatsu et al. [30,31,32], Xiao et al. [33,34,35], Takase et al. [36,37,38], and Salam et al. [39,40,41]. These studies promote a better understanding of the interaction between plants and their environment, offering a range of innovative solutions for achieving higher yields while maintaining a sustainable environment.
